# An Audience Facilitates Facial Feedback: A Social-Context Hypothesis Reconciling Original Study and Nonreplication

**DOI:** 10.1177/00332941231153975

**Published:** 2023-02-03

**Authors:** R. Hans Phaf, Mark Rotteveel

**Affiliations:** Amsterdam Brain and Cognition Center, 100440 Universiteit van Amsterdam, Amsterdam, Netherlands; Brain and Cognition Group, Department of Psychology, Universiteit van Amsterdam, Amsterdam, Netherlands; Amsterdam Brain and Cognition Center, 1234 Universiteit van Amsterdam, Amsterdam, Netherlands; Social Psychology Program, Department of Psychology, 1234 Universiteit van Amsterdam, Amsterdam, Netherlands

**Keywords:** Facial feedback, audience effects, social context, video awareness, theoretical analysis

## Abstract

Nonreplications of previously undisputed phenomena tend to leave a theoretical vacuum. This theoretical perspective seeks to fill the gap left by the failure to replicate unobtrusive facial feedback. In the emblematic original study, participants who held a pen between the teeth (i.e., requiring activity of the zygomaticus major muscle) rated cartoons more positively than participants who held the pen between the lips. We argue that the same social mechanisms (e.g., the presence of an audience) modulate facial feedback to emotion as are involved in the feed-forward shaping of facial actions by emotions. Differing social contexts could thus help explain the contrast between original findings and failures to obtain unobtrusive facial feedback. An exploratory analysis that included results only from (unobtrusive) facial-feedback studies without explicit reference to emotion in the facial manipulation provided preliminary support for this hypothesis. Studies with a social context (e.g., due to experimenter presence) showed a medium-sized aggregate facial-feedback effect, whereas studies without a social context (e.g., when facial actions were only filmed), revealed a small effect. Video awareness strengthened facial feedback considerably within an engaging social context, but seemed to reduce it without a social context. We provisionally conclude that a (pro-)social interpretation of facial actions facilitates feedback to (primarily positive) emotion, and suggest further research explicitly manipulating this context.

Does the face represent the “royal road to the emotions” (p. 388; Crivelli & Fridlund, 2018)? Following Darwin (1872), a consensus has grown that a limited number of facial action patterns (“expressions”) have been evolutionarily inherited and represent human universals (e.g., Pascalis & Kelly, 2009). The face appears to have evolved to emit decorrelated signal patterns that can be easily distinguished from each other by an observer (cf. Smith et al., 2005). However, the relationship between these facial-action patterns and emotions remains hotly debated (e.g., Shariff & Tracy, 2011). In the most extreme position, facial expressions are viewed as involuntary manifestations of internal mental states (i.e., emotions) of which only the varnish can change through culturally different display rules (see Ekman, 1993). This position entails a one-to-one relationship between universal facial expressions and equally universal, basic emotions, which can operate in both the feed-forward and feedback directions, and should be evident not only in the production but also in the recognition of emotion-specific expressions. Receiver participants, for instance, who watched spontaneously produced (i.e., not posed), and covertly videotaped, facial expressions of other participants in response to slides with emotional content, were able, above chance, to categorize these expressions correctly. This was especially true for happy, angry, and disgust expressions, but not for surprise expressions (Wagner et al., 1986). Alternatively, a less tight connection between facial actions and emotion may exist, as reflected, for instance, in the behavioral ecology view proposed by Fridlund (1994). He posits that facial expressions reflect intentions in social interactions rather than emotional meanings per se. After first having introduced the facial-feedback hypothesis, we will argue that not only in the feed-forward but also in the feedback direction social interpretations modulate the link between emotions and facial actions.

## Facial Feedback

It is only a small step to assume bidirectionality if the relationship between facial expressions and internal emotions is indeed one-to-one. Specific facial actions could be able to strengthen, or even initiate, the corresponding emotion within the expressor (i.e., through facial feedback). Darwin (1872) noted that “The free expression by outward signs of an emotion intensifies it. On the other hand, the repression, as far as this is possible, of all outward signs softens our emotions... Even the simulation of an emotion tends to arouse it in our minds” (p. 366). However, to conclude to such a relationship in experimental research, expression effects should be investigated in total isolation from other emotional influences, contexts, or interpretations. *Strack et al. (1988) approximated this situation in their seminal study by an unobtrusive manipulation of facial actions (i.e., holding a pen between the lips or between the teeth) and by providing a non-emotional cover story (i.e., simulating physically impaired persons who use their mouth to write). These manipulations were intended to prevent the participants from interpreting these facial actions in emotional terms, for instance as a smile when holding the pen between the lips. In accordance with the facial-feedback hypothesis, participants who held a pen between the teeth (i.e., requiring zygomaticus major muscle activity) during the rating of cartoons felt more amused by the stimuli than participants who held the pen between the lips (i.e., zygomaticus major muscle activity is inhibited).

## A Nonreplication

A large multi-lab replication attempt (1894 participants) failed to replicate the pen-in-mouth effect and cast doubt on the facial-feedback hypothesis (Wagenmakers et al., 2016). Although the attempt closely mimicked the original setup of Strack and collaborators (*Strack et al., 1988), the replicators deviated in four respects from the original study. New cartoons were selected, experimenter-participant interactions were minimized, the task was recorded by a video camera, and the cartoon rating only targeted the emotional component of the humor response (cf. Study 2 of Strack et al.). There has been much debate about whether some or all of these deviations are critical for obtaining the facial-feedback findings (Strack, 2016, 2017; Stroebe, 2019). Wagenmakers and collaborators, however, focused on the reproducibility of the data but did not elaborate on the theoretical implications of their nonreplication (cf. Strack, 2016). Hence, they could not appraise the impact of these deviations. Of course, it remains possible that the nonreplication has no theoretical implications, and that it resulted from meaningless fluctuations or hidden variations in trivial moderators. Alternatively, such fluctuations and variations may have caused the original findings in the first place. More importantly, a meaningful moderator, unknown to both the original authors and the replicators, may have been instrumental in the disparate findings. The current state of theoretical indecision seems highly unsatisfactory and should be resolved with further experimentation or analysis.

## Overcoming the Theoretical Indecision

Recently, Coles et al. (2019) conducted a comprehensive meta-analysis across a large number of different facial feedback studies to estimate an aggregate effect size and to contrast the findings for different levels of theoretically motivated moderator variables. Meta-analysis essentially attempts to extend estimation to more than one study (e.g., combining original study and replication study) and integrates evidence from multiple studies to increase precision (Cumming, 2014). More importantly, meta-analysis can stimulate theoretical development by allowing studies to be rearranged along new moderator variables to examine a new hypothesis even before it is actually investigated experimentally. Coles and collaborators searched for published and unpublished, English-language articles that manipulated facial actions (not simultaneously manipulated body postures), collected measures of emotional experience or affective judgments, tested non-clinical samples, and provided sufficient information to calculate effect sizes. Eventually, 286 effects from 138 different facial-feedback studies were selected including explicit emotion regulation (i.e., by overt facial pose manipulation) studies, and more implicit (i.e., unobtrusive) facial manipulation studies. Overall, a small and highly variable effect of facial feedback emerged (Cohen’s *d* = 0.20; 95% CI [0.14, 0.26]), with scant evidence of publication bias. In addition, distinguishing twelve potential moderator variables offered few clues for explaining the discrepancy between original study and nonreplication. Awareness of the facial feedback manipulation did not seem to matter much (aware *d* = 0.15; 95% CI [0.06, 0.24]; 67 studies; unaware *d* = 0.13; 95% CI [−0.05, 0.31]; 14 studies).

A potential explanation for the nonreplication by Wagenmakers et al. (2016) was put forward by Strack (2016). He suggested that the video recording in the nonreplication had induced a subjective self-focus, disrupting the flow of experience and suppressing emotional responses. The video camera that Wagenmakers and collaborators (2016) used would make participants self-conscious and therefore less prone to express their emotions. Coles et al. (2019) explicitly addressed this explanation for the discrepancy by including awareness of video recording as a moderator in their meta-analysis. Ethical and privacy considerations often prompt researchers to forewarn the participant about these recordings (i.e., 54 studies). Other studies only informed participants afterwards or had no video camera present (i.e., 73 studies). Very little evidence emerged that the effect was smaller when participants were aware of the recording (Cohen’s *d* = 0.17; 95% CI [0.06, 0.28]) than when they were unaware or no video camera was present (*d* = 0.24; 95% CI [0.15, 0.32]). Interestingly, Strack’s self-focus hypothesis introduces negative social interpretations as a factor in facial feedback. Conversely, could prosocial interpretations enhance facial feedback?

## Facial Actions as Social Tools

Theorizing about the feed-forward effects of emotions on facial actions in terms of social context suggests that this factor may also account for the discrepancy in facial-feedback findings. The behavioral ecology view of Fridlund (1994; see also Crivelli & Fridlund, 2018) holds that facial morphologies are not intrinsically tied to emotional meanings but are used as “social tools” displaying our intentions in interactions with other members of our species. They evolved to communicate the motives of an expressor in a particular social situation. The smile, for instance, which may have originated from a fearful protective action (i.e., baring the teeth when facing aggressive challenges; cf. Preuschoft & van Hooff, 1997), has become a sign of friendly intentions (i.e., by showing that “I am afraid, so I am not a threat”).

Social contexts have often been found to facilitate facial expression of emotions. Participants show easily recognizable facial expressions when smelling pleasant, neutral, and unpleasant odors if they know a human observer is present, but if those same odors are smelled without someone watching, and faces are secretly filmed, fewer expressions can be seen (Gilbert et al., 1987; for comparable audience effects in infants and adults, see Chovil, 1991; Jones et al., 1991; Kraut & Johnston, 1979; Ruiz-Belda et al., 2003). In contrast to the disparate facial expressions, similar emotional evaluations were made of the strong odors in the observer-present and video-recorded conditions. Apparently, the magnitude of emotions was similar in both conditions, but facial actions were modulated by human presence or absence. The physical presence of a person in the room seems not even to be required for these audience effects, and an imagined sociality of the situation may suffice (e.g., Fridlund et al., 1990). A social interpretation thus seems to be crucial in the feed-forward link between emotions and facial expressions.

If audience effects work in the feed-forward direction, they may also be expected to influence the feedback connection between facial actions and emotions. The presence of another person may convey a social interpretation to these facial actions that otherwise seem to lack meaning. A smile produced in a social context, for instance, may signal a polite greeting in anticipation of positive interactions. In many cases, such as in studies of emotion regulation via facial feedback included in the meta-analysis of Coles et al. (2019), subtle context factors may be overridden by the interpretation explicitly provided in the instructions. However, when facial actions are unobtrusively manipulated, their emotional interpretation may depend heavily on additional context factors. The original facial-feedback study (*Strack et al., 1988) and the nonreplication (Wagenmakers et al., 2016) differed along the same dimension (i.e., observer present vs. expressions being only filmed) as with the audience manipulation of Gilbert et al. (1987). Facial feedback may have been facilitated by the social context of the experimenter’s presence in the original study. One could thus argue that Strack and collaborators have not fully succeeded in isolating facial actions from other emotional factors, contexts or interpretations. We hypothesize here that in the absence of explicit cues, the facial movements will only be experienced as emotional when interpreted in a social context.

## Exploratory Analysis

To investigate the audience hypothesis for facial feedback, we rearranged (i.e., repartitioned) a subset of the studies in the meta-analysis by Coles and collaborators (2019) along the novel moderator of either a social context being present or absent during the facial manipulation (see Appendix A). Only the unobtrusive facial feedback studies (i.e., without an explicit reference) from Coles et al. (2019) were included in this subset. If an explicit reference is made, the subtle influence of an audience, either the experimenter or other participants in a group setting, can be overwhelmed by the instructions that link the facial actions to emotions. The subset was also subdivided along the video-awareness moderator of Coles and collaborators. Consistent with the audience hypothesis, the presence of a social context resulted in unobtrusive facial-feedback effects at medium levels, whereas without a social context, they remained at low levels (see Appendix A). Similar medium-sized unobtrusive facial-feedback effects were obtained by Marsh et al. (2019) in a large-scale classroom study (i.e., with social context), which was performed after the nonreplication of Wagenmakers et al. (2016) and the meta-analysis of Coles et al. (2019). When the social-context moderator was subdivided along the video-awareness moderator, the self-focus hypothesis (see Strack, 2016) only received limited support in the studies without social context, but was clearly contradicted in the studies with social context. The unobtrusive facial-feedback effect with video-aware participants even approached large effect sizes in the latter studies.

The exploratory analysis provisionally indicates that a social context, or at least one inferred by the participants, modulates the emotional consequences of facial actions (cf. Hess, 2021). Social smiling in such a context, for instance, may be facilitated or inhibited by the pen between the teeth or lips, respectively. The results of this analysis do not support the sufficiency hypothesis (see Hess et al., 1992), which postulates that performing facial actions unobtrusively in the absence of other emotionally salient cues may be sufficient to elicit an emotional experience. Facial feedback appears to contribute to, rather than be a causal factor in, emotional experience. In contrast, Coles et al. (2019) obtained more evidence for facial actions as an initiator (*d* = 0.32; 95% CI [0.15, 0.49]; 28 studies) than as a modulator (*d* = 0.13; 95% CI [0.07, 0.18]; 93 studies) of affect. However, this may be because their meta-analysis combined many different types of studies, some of which also attempted to explicitly regulate emotion through facial manipulation. The presence of other persons rather than the facial actions per se may have unwittingly served as the initiator in studies supporting that facial actions evoke emotion. A potential confound of the initiation-modulation moderator and the social-context variable may have gone unnoticed by Coles and associates because they did not recognize the importance of social context. In any case, when the inclusion is limited to studies without an explicit reference to emotion in the facial manipulation, an appreciably larger effect emerges with a social context than overall in the meta-analysis of Coles and collaborators.

## The Social Context in Experimental Research

Facilitation of facial feedback by social context is probably not limited to passive audience effects, but may take a more active form, such as in formalized interactions with the experimenter. Smiling is certainly part of the culturally universal greeting response (i.e., together with raising the eyebrows; cf. Eibl-Eibesfeldt, 2007). A conversation with another person without social smiling is deemed highly unnatural and may even be considered dismissive (cf. Hess, 2021). Compared to when experimental instructions are only presented on a computer screen, a smiling experimenter fosters greater levels of cooperation and may involve fewer unmotivated participants (cf. Johnston et al., 2010). The percentage of participants who are unwilling or unmotivated to comply with instructions, which anecdotally can be as high as 40% in some experiments, often seems to be severely underestimated, especially in replication attempts. In addition to social smiling, there is evidence that people automatically mimic the facial expressions they perceive and that this mimicry can help them recognize the emotions of others (e.g., Wood et al., 2016). Holding the pen between the lips could suppress a mimicked smile and inhibit a positive experience associated with the social interaction.

Experimenter bias may be another explanation for the larger facial-feedback effects in a social context (cf. Rosenthal & Fode, 1963). The implicit expectation in favor of facial feedback emanating from the experimenter could have influenced the outcomes in some unknown manner. Wagenmakers et al. (2016) probably wanted to minimize this type of bias by recording the participants’ faces with a video camera, while the experimenter was not present in the experimental room. Interestingly, Doyen et al. (2012) in their replication attempt of the behavioral priming study of Bargh et al. (1996) found that the direction of experimenter expectations was crucial in obtaining the priming effect. In these experiments, walking speed was measured after unwittingly exposing participants to stereotypes of old age. Doyen and collaborators measured walking speed both “subjectively” (i.e., by a handheld stopwatch as in the original study) and “objectively” (i.e., by an infrared timing device). The slowing of walking speed in both measurements, when experimenters were led to believe that the behavioral priming should occur, demonstrated that the speed measurements were not biased by the experimenter per se but that expectations may have been implicitly transferred to the participants. Nonverbal communication between experimenter and participant, perhaps in the form of subtle facial or postural expressions, may thus have been responsible for the adoption of the elderly behavior by the latter and appears to have a stronger influence than the elderly stereotype priming. The present exploratory analysis indicates that experimenter presence also enhances facial feedback. We conjecture here that it arises either through facilitation/inhibition of mimicked experimenter smiles or from a more indirect (pro-)social interpretation of the facial actions induced by the presence of an experimenter or other persons. Rather than as an alternative interpretation for the facial-feedback effect, experimenter bias is considered here as a member of a more general group of social-context hypotheses.

## Video Awareness Interacts with Social Context

Further indications that facial feedback is strongly influenced by the participant’s interpretation of the situation were found in the opposite directions of these effects due to the video-awareness moderator in the social context present/absent conditions. Appraisal theories of emotion involving evaluative interpretations of situations (e.g., Frijda, 1986) have already been implicated in feed-forward effects on facial actions (cf. Hess & Thibault, 2009), and may also be invoked to explain facial-feedback effects. The awareness of being monitored by an experimenter in another room seems to be appraised differently than direct contact with such an experimenter. Impersonal instructions delivered on a screen telling participants that their faces are filmed could easily induce a “big brother is watching you” appraisal and an inhibition of facial feedback by an increased self-focus (cf. Strack, 2016). Conversely, a friendly and nearby experimenter introducing the experiment and explaining the purpose of the video camera would elicit a more prosocial interpretation that seems to enhance facial-feedback effects. A remaining question is what role experimenter expectations play in this social context, whether subtle clues about the expectations are transferred to the participant or whether facial actions are conveyed more directly from experimenter to participant, for instance through mimicking.

## Conclusion

Based on this theoretical analysis, no definitive statements can be made about the role of social context or experimenter bias. Although receiving further support from later experimental results (Marsh et al., 2019) not included in the meta-analysis of Coles et al. (2019), our exploratory analysis is not sufficient for drawing firm conclusions. New unobtrusive facial feedback experiments are required that explicitly manipulate social context, for instance, with naïve experimenters or even experimenters instructed against the facial feedback hypothesis, with cooperative versus dismissive experimenters, or group versus solitary setups. Social context should not be considered a nuisance variable that is to be minimized, but rather should be incorporated explicitly in theorizing and experimentation. Enhanced methodological scrutiny and more rigorous control by isolating participants into cubicles and running computerized experiments seems a natural response to experimenter bias and demand characteristics, but may have the unwanted side effect of unwittingly altering the processes studied. We fully agree with Klein et al. (2012): “In conclusion, we should like to recommend that rather than attempting to eliminate all potential sources of bias by automatizing experiments, experimental psychologists should instead strive to explore such sources of bias in a systematic manner” (p.582). It must also be acknowledged that there remain many “unknown unknowns” that can plague researchers and replicators alike. Fiedler et al. (2012) already noted that many theoretical hypotheses run the risk of being prematurely rejected by seemingly contradictory experimental data obtained in suboptimally calibrated conditions due to a lack of knowledge about the best stimuli, task settings, and context conditions. Theoretical analyses like the present one may be very helpful in discovering these hidden moderators, and in this case, support the provisional notion that the appraisal of the social context is a crucial factor in facial feedback.

## Appendix A

The repartitioning of studies from the meta-analysis of Coles et al. (2019; materials available from https://osf.io/v8kxb/), was performed with the classical meta-analysis option of the open-source statistical program JASP (https://jasp-stats.org/). A subset of the effect sizes (Cohen’s *d*) collected by Coles and colleagues in their literature search through 2017 was subjected to a random-effects analysis using the DerSimonian-Laird method (see Grasman, 2017). All other study properties and moderator divisions (e.g., awareness of video recording) were copied from Coles and colleagues. The *p*-values are only provided here for illustrative purposes because all tests were conducted post-hoc.

The studies from the meta-analysis of Coles et al. (2019) were scored on two variables; reference to emotion in the facial manipulation instructions, and social context. Only (unobtrusive) studies in which the participants had no explicit cues for emotionally interpreting their facial actions (i.e., no reference to emotion) were included. In the other studies, subtle contextual factors could be overwhelmed, for instance, by explicit instructions to regulate emotions through facial movements. Facial manipulations of surprise (e.g., Reisenzein & Studtmann, 2007) were also excluded because the status of surprise as an emotion is uncertain (cf. Wagner et al., 1986). The present selection consisted of *k* = 136 effect sizes from *s* = 72 studies not explicitly referring to emotion in the facial manipulation, which were further partitioned on the social-context moderator (see Table A1). The only study to specifically manipulate social context was by *Miyamoto (2006) and these effect sizes were scored accordingly. If in the other studies, an experimenter or other participants were physically present in the experimental room, this was scored as social context present. An experimenter present only during the initial facial instructions could also set up a social interpretation of the face manipulation and thus was scored as social context present (e.g., *Hawk et al., 2012). The multi-lab replication study of Wagenmakers et al. (2016), however, in which the facial instructions were delivered through a video on a computer screen and participants were filmed, was scored as social context absent.

**Table A1.** Studies without reference to emotion in the facial manipulation selected from the meta-analysis of Coles et al. (2019; experimental characteristics and effect sizes from https://osf.io/v8kxb/), which were classified on the social-context moderator.

Study	Awareness of Recording	Social Context	Design	*N*	*d*
Andréasson (2010) Study 3	No	Yes	Within	48	−0.05
Andréasson (2010) Study 3	No	Yes	Within	48	−0.35
Andréasson (2010) Study 4	No	Yes	Within	44	0.49
Andréasson (2010) Study 4	No	Yes	Within	44	0.31
*Andréasson and Dimberg (2008)	No	Yes	Between	112	−0.22
*Baumeister et al. (2016)	No	Yes	Within	10	1.26
*Baumeister et al. (2016)	No	Yes	Within	10	0.63
*Bodenhausen et al. (1994)	No	Yes	Between	51	0.55
*Bush et al. (1989)	No	No	Between	69	0.16
*Davey et al. (2013) Study 1	No	Yes	Between	28	0.41
*Davey et al. (2013) Study 1	No	Yes	Within	14	0.62
*Davey et al. (2013) Study 1	No	Yes	Between	28	0.52
*Davey et al. (2013) Study 1	No	Yes	Within	14	0.13
*Davey et al. (2013) Study 1	No	Yes	Between	28	0.69
*Davey et al. (2013) Study 1	No	Yes	Within	14	0.42
*Davey et al. (2013) Study 1	No	Yes	Between	28	0.35
*Davey et al. (2013) Study 1	No	Yes	Within	14	0.14
*Davey et al. (2013) Study 2	No	Yes	Between	29	0.73
*Davey et al. (2013) Study 2	No	Yes	Within	15	0.63
*Davey et al. (2013) Study 2	No	Yes	Between	29	0.4
*Davey et al. (2013) Study 2	No	Yes	Within	15	0
*Davey et al. (2013) Study 2	No	Yes	Between	29	0.08
*Davey et al. (2013) Study 2	No	Yes	Within	15	−0.25
*Davey et al. (2013) Study 2	No	Yes	Between	29	0.03
*Davey et al. (2013) Study 2	No	Yes	Within	15	−0.06
*Davis et al. (2010)	No	No	Between	68	0.1
*Davis et al. (2010)	No	No	Between	68	0.05
*Davis et al. (2010)	No	No	Between	68	−0.15
*Davis et al. (2009)	No	No	Between	69	0.07
*Davis et al. (2009)	No	No	Between	69	0.51
*Davis et al. (2015)	No	No	Within	18	−0.16
*Dimberg and Söderkvist (2011) Study 1	No	No	Within	48	0.51
*Dimberg and Söderkvist (2011) Study 2	No	No	Within	96	0.1
*Dimberg and Söderkvist (2011) Study 2	No	No	Within	96	0.32
*Dimberg and Söderkvist (2011) Study 3	No	No	Within	61	0.06
*Dimberg and Söderkvist (2011) Study 3	No	No	Within	61	0.31
*Dimberg and Söderkvist (2011) Study 3	No	No	Within	61	0.34
*Duncan and Laird (1980)	No	Yes	Within	60	0.59
*Duncan and Laird (1980)	No	Yes	Within	60	0.44
*Dzokoto et al. (2014)	No	Yes	Between	70	1.02
*Dzokoto et al. (2014)	No	Yes	Between	59	0.07
*Dzokoto et al. (2014)	No	Yes	Between	35	1.07
*Dzokoto et al. (2014)	No	Yes	Between	51	0.2
*Flack (2006)	Yes	Yes	Within	51	0.72
*Flack (2006)	Yes	Yes	Within	51	0.35
*Flack (2006)	Yes	Yes	Within	51	0.59
*Flack (2006)	Yes	Yes	Within	51	0.68
*Flack et al. (1999a) Study 1	Yes	Yes	Within	60	1.2
*Flack et al. (1999a) Study 1	Yes	Yes	Within	60	0.7
*Flack et al. (1999a) Study 1	Yes	Yes	Within	60	0.31
*Flack et al. (1999a) Study 1	Yes	Yes	Within	60	0.86
*Flack et al. (1999a) Study 1	Yes	Yes	Within	60	1.31
*Flack et al. (1999a) Study 2	Yes	Yes	Within	29	0.39
*Flack et al. (1999a) Study 2	Yes	Yes	Within	29	0.23
*Flack et al. (1999a) Study 2	Yes	Yes	Within	29	−0.16
*Flack et al. (1999a) Study 2	Yes	Yes	Within	29	−0.49
*Flack et al. (1999a) Study 2	Yes	Yes	Within	29	0.25
*Flack et al. (1999b)	Yes	Yes	Within	54	1.41
*Flack et al. (1999b)	Yes	Yes	Within	54	0.29
*Flack et al. (1999b)	Yes	Yes	Within	54	1.18
*Flack et al. (1999b)	Yes	Yes	Within	54	1.21
*Hawk et al. (2012)	No	Yes	Between	41	0.85
*Helt and Fein (2016)	NA	Yes	Within	43	0.42
*Ito et al. (2006)	No	Yes	Within	40	−0.39
*Ito et al. (2006)	No	Yes	Between	33	−0.25
*Laird (1974) Study 1	No	Yes	Within	38	0.46
*Laird (1974) Study 1	No	Yes	Within	38	0.44
*Laird (1974) Study 1	No	Yes	Within	38	0.39
*Laird (1974) Study 2	No	Yes	Within	26	0.55
*Laird (1974) Study 2	No	Yes	Within	26	0.13
*Laird and Crosby (1974) Study 1	No	Yes	Within	26	−0.13
*Laird and Crosby (1974) Study 2	No	Yes	Within	26	0.35
*Larsen et al. (1992)	No	Yes	Within	27	0.43
*Lewis (2012)	No	Yes	Within	24	0.71
*Lewis (2012)	No	Yes	Within	24	0.56
*Marmolejo-Ramos and Dunn (2013) Study 1	No	No	Within	100	−0.07
*Marmolejo-Ramos and Dunn (2013) Study 2	No	No	Within	106	−0.07
*Marmolejo-Ramos and Dunn (2013) Study 3	No	No	Within	104	−0.07
*Marmolejo-Ramos and Dunn (2013) Study 4	No	No	Within	100	−0.07
*Marmolejo-Ramos and Dunn (2013) Study 5	No	No	Within	66	0.27
*Marmolejo-Ramos and Dunn (2013) Study 6	No	No	Within	67	0.38
*McIntosh et al. (1997)	No	Yes	Within	26	0.54
*Meeten et al. (2015)	No	Yes	Within	71	0.49
*Miyamoto (2006) Study 1	No	No	Between	40	0.17
*Miyamoto (2006) Study 1	No	Yes	Between	40	0.53
*Paredes et al. (2013)	No	Yes	Between	31	0.85
*Roemer (2014)	Yes	Yes	Between	44	0.58
*Roemer (2014)	Yes	Yes	Between	44	0.29
*Rummer et al. (2014)	No	Yes	Between	74	0.57
*Rummer et al. (2014)	No	Yes	Between	74	0.46
*Söderkvist and Dimberg (2021)	No	No	Within	32	0.36
*Söderkvist et al. (2018) Study 1	No	No	Within	32	0.34
*Söderkvist et al. (2018) Study 2	No	No	Within	64	0.17
Soussignan (2002)	Yes	Yes	Between	33	−0.17
Soussignan (2002)	Yes	Yes	Between	33	0.48
Soussignan (2002)	Yes	Yes	Between	33	0.47
Soussignan (2002)	Yes	Yes	Between	33	0.44
Soussignan (2002)	Yes	Yes	Between	32	0.53
Soussignan (2002)	Yes	Yes	Between	32	1.1
Soussignan (2002)	Yes	Yes	Between	32	1.11
Soussignan (2002)	Yes	Yes	Between	32	0.94
*Stel et al. (2008) Study 3	No	Yes	Between	24	1
*Strack et al. (1988) Study 1	No	Yes	Between	76.67	0.43
*Strack et al. (1988) Study 2	No	Yes	Between	83	−0.15
*Strack et al. (1988) Study 2	No	Yes	Between	41.5	0.55
*Strack et al. (1988) Study 2	No	Yes	Between	41.5	−0.51
*Tamir et al. (2004)	No	Yes	Between	72	−0.16
*Tourangeau and Ellsworth (1979)	Yes	No	Between	20.5	0.3
*Tourangeau and Ellsworth (1979)	Yes	No	Between	20.5	0.3
*Tourangeau and Ellsworth (1979)	Yes	No	Between	20.5	0.3
*Tourangeau and Ellsworth (1979)	Yes	No	Between	20.5	0.3
*Trent (2010)	No	No	Between	107.33	−0.22
*Trent (2010)	No	No	Between	107.33	−0.22
*Trent (2010)	No	No	Between	107.33	−0.06
*Trent (2010)	No	No	Between	107.33	−0.06
Wagenmakers et al. (2016) Albohn site	Yes	No	Between	139	0.09
Wagenmakers et al. (2016) Allard site	Yes	No	Between	125	0.09
Wagenmakers et al. (2016) Benning site	Yes	No	Between	115	−0.01
Wagenmakers et al. (2016) Bulnes site	Yes	No	Between	101	0.09
Wagenmakers et al. (2016) Capaldi site	Yes	No	Between	117	−0.07
Wagenmakers et al. (2016) Chasten site	Yes	No	Between	94	−0.04
Wagenmakers et al. (2016) Holmes site	Yes	No	Between	99	0.15
Wagenmakers et al. (2016) Koch site	Yes	No	Between	100	−0.14
Wagenmakers et al. (2016) Korb site	Yes	No	Between	101	0.01
Wagenmakers et al. (2016) Lynott site	Yes	No	Between	126	0.23
Wagenmakers et al. (2016) Oosterwijk site	Yes	No	Between	110	−0.17
Wagenmakers et al. (2016) Ozdogru site	Yes	No	Between	87	−0.3
Wagenmakers et al. (2016) Pacheco-Unguetti site	Yes	No	Between	120	−0.08
Wagenmakers et al. (2016) Talarico site	Yes	No	Between	112	0.02
Wagenmakers et al. (2016) Wagenmakers site	Yes	No	Between	130	0.13
Wagenmakers et al. (2016) Wayand site	Yes	No	Between	110	−0.14
Wagenmakers et al. (2016) Zeelenberg site	Yes	No	Between	108	0.25
*Zajonc et al. (1989) Study 3	No	Yes	Within	37	1.27
*Zajonc et al. (1989) Study 4	NA	Yes	Within	26	0.47
*Zajonc et al. (1989) Study 4	NA	Yes	Within	26	0.31
*Zhu et al. (2015)	Yes	Yes	Between	55	1.74

The *s* = 36 studies and *k* = 88 effect sizes with social context resulted in an aggregate effect size of *d* = 0.444, 95% CI [0.349, 0.539], *z* = 9.176 *p* < 0.001, with a large remaining amount of heterogeneity (*τ*^*2*^ = 0.163; *l*^*2*^ = 91.000). The *s* = 36 studies and *k* = 48 effect sizes without social context had an overall effect size of *d* = 0.099, 95% CI [0.032, 0.162], *z* = 3.066 *p* = 0.002, with still a considerable amount of heterogeneity (*τ*^*2*^ = 0.024; *l*^*2*^ = 62.522). The presence of a social context enhanced facial feedback, β = 0.352, 95% CI [0.217, 0.487], *p* < 0.001. Egger’s tests showed no clear indication for publication bias at either moderator level, with social context, *z* = 1.401, *p* = 0.161; without social context, *z* = −0.985, *p* = 0.325.

Strack’s (2016) hypothesis of self-focus due to awareness of video recording was examined here only in the unobtrusive studies (i.e., without explicit reference to emotion in the face manipulation). The subdivision by Coles et al. (2019) into studies with and without awareness for video recording was applied to the present selection (see Table A1). They scored studies that did not have a video camera for face recording as unaware of video recording. The studies that had a camera present but reported no details about the forewarning of this presence were scored as N/A by these authors. The latter studies were also excluded here from this video-awareness moderator. In accordance with the self-focus hypothesis, facial feedback effects in the absence of a social context tended to be smaller with awareness, *d* = 0.026, 95% CI [-0.062, 0.115], *k* = 20, *z* = 0.584 *p* = 0.559, than without awareness of video recording, *d* = 0.129, 95% CI [0.045, 0.213], *k* = 28, *z* = 3.005 *p* = 0.003; β = −0.103, 95% CI [-0.237, 0.032], *p* = 0.136. Interestingly, a reverse pattern of effects emerged in the studies with social context. Facial-feedback effects in the presence of a social context were much larger with video awareness, *d* = 0.629, 95% CI [0.434, 0.825], *k* = 29, *z* = 6.303 *p* < 0.001, than without awareness, *d* = 0.344, 95% CI [0.240, 0.449], *k* = 56, *z* = 6.452 *p* < 0.001; β = 0.276, 95% CI [0.068, 0.483], *p* = 0.009. Contrary to the self-focus hypothesis, facial-feedback effects, therefore, were strengthened considerably by the awareness of the video recording when embedded in a social context.
